# CMR评估肺高血压：肺高血压患者与健康志愿者对照研究

**DOI:** 10.3779/j.issn.1009-3419.2016.05.07

**Published:** 2016-05-20

**Authors:** 蒙 王, 振文 杨, 璋 张, 东 李, 帆 杨, 铁链 于

**Affiliations:** 1 300052 天津，天津医科大学总医院放射科 Department of Radiology, Tianjin Medical University General Hospital, Tianjin 300052, China; 2 300052 天津，天津医科大学总医院心内科 Department of Cardiovascular Disease, Tianjin Medical University General Hospital, Tianjin 300052, China

**Keywords:** 心脏, 核磁共振, 肺高血压, 右心室, 肺动脉, Cardiac, Magnetic resonance, Pulmonary hypertension, Right ventricle, Pulmonary artery

## Abstract

**背景与目的:**

肺高血压（pulmonary hypertension, PH）临床病程表现为进行性恶化和间断急性失代偿发作，死亡可突然或缓慢发生，而临床医生往往对病情的进展评估不足。本研究旨在应用心脏核磁共振（cardiac magnetic resonance, CMR）评估PH患者右心室（right ventricle, RV）形态、功能及主肺动脉（main pulmonary artery, MPA）血液动力学，并与健康人对照，探讨CMR在检出PH中的临床应用价值。

**方法:**

对56例PH患者及22例健康志愿者进行CMR扫描并获得RV舒张末期容积（end-diastolic volume, EDV）、收缩末期容积（end-systolic volume, ESV）、射血分数（ejection fraction, EF）、心肌质量（myocardial mass, MM）、RV面积变化分数（RV fractional area change, RVFAC）、室间隔与左室游离壁曲率比，MPA正向峰值流速、最大截面积、最小截面积及顺应性。PH组与对照组测量结果采用两独立样本t检验进行比较。受试者工作特征曲线（receiver operating characteristic curve，ROC曲线）用于比较MPA正向峰值流速、顺应性、曲率比、RVFAC单项指标及综合评估检出PH的效能。*P* < 0.05为差异有统计学意义。

**结果:**

与对照组相比，PH组RV形态改变、功能明显下降，血液动力学指标恶化。顺应性对检出PH的效能较高（AUC为0.950）；综合应用正向峰值流速、顺应性、曲率比和RVFAC时，检出PH的效能最高（AUC为0.988）。

**结论:**

CMR综合评估指标有利于准确反映PH患者RV-肺循环整体状态。

## Abstract (en)

**Background and objective:**

The clinical course of pulmonary hypertension (PH) is one of progressive deterioration interspersed with episodes of acute decompensation. It is difficult to predict when patients will die because death may come either suddenly or slowly due to progressive heart failure. The aim of this study is to investigate morphology, function and hemodynamics in PH, compared with healthy people, and to investigate the clinical value of detection of PH by use of cardiac magnetic resonance (CMR) parameters.

**Methods:**

CMR was performed in 56 PH patients collected from Tianjin Medical University General Hospital from January 2012 to December 2014 and 22 healthy controls. The following parameters were calculated: right ventricle (RV) end-diastolic volume (EDV), end-systolic volume (ESV), ejection fraction (EF), myocardial mass (MM), RV fractional area change (RVFAC), interventricular septal curvature (C_IVS_), left ventricular free wall curvature (C_FW_), and C_IVS_/C_FW_, main pulmonary artery (MPA) positive peak velocity, maximal area, minimal area and distensibility. Comparisons of CMR measurements between PH patients and controls were analyzed by using the student t-tests. Receiver operating characteristic (ROC) curve analysis was used to compare the PH diagnostic abilities for four parameters (MPA positive peak velocity, distensibility, curvature ratio, and RVFAC) and combined CMR parameter. *P* < 0.05 was considered significant.

**Results:**

Compared with healthy controls, RV morphology, function and hemodynamics of PH group declined and deteriorate obviously. The ROC curve analysis showed that among the four parameters distensibility of MPA had the highest AUC value (AUC=0.95). Additionally, combined CMR parameter (positive peak velocity+distensibility+curvature ratio+RVFAC) had even higher AUC (AUC=0.988).

**Conclusion:**

Comprehensive CMR parameters is conducive to accurately reflect the overall state RV-pulmonary circulation in patients with PH.

肺高血压（pulmonary hypertension, PH）是一种可见于多种临床疾病，并将使大多数病例的心血管和呼吸系统疾病更难控制的血液动力学和病理生理学异常状态，其主要特征是肺动脉压力和肺血管阻力进行性升高，肺循环广泛性血管壁细胞性增殖和重构^[1]^。PH临床病程表现为进行性恶化和间断急性失代偿发作，死亡可突然或缓慢发生，而临床医生往往对病情的进展评估不足^[1, 2]^。心脏核磁共振（cardiac magnetic resonance, CMR）对PH的诊断、分类、失代偿危险评估、疗效随访和判断预后都具有应用潜力^[3]^。本研究旨在应用CMR评估一组PH患者右心室（right ventricle, RV）形态、功能及主肺动脉（main pulmonary artery, MPA）血液动力学，并与健康人对照，探讨CMR在检出PH中的临床应用价值。

## 1 资料与方法

### 1.1 研究对象

根据PH临床诊断标准^[1]^，收集天津医科大学总医院2012年1月-2014年12月期间经右心导管（right heart catheterization, RHC）确诊的PH患者共56例，女性51例，年龄（38.93±11.83）岁，心率（81.45±9.80）次/分，均为毛细血管前PH。56例患者中肺动脉高压（pulmonary arterial hypertension, PAH）50例，其中特发性PAH、结缔组织病相关性PAH、先天性心脏病相关性PAH分别为10例、27例、13例；慢性血栓栓塞性肺高血压（chronic thromboembolic pulmonary hypertension, CTEPH）6例。健康志愿者22例，女性20例，年龄（37.15±12.94）岁，心率（72.95±6.02）次/分，年龄和性别的构成比均与PH组相匹配，心率、血压均在正常范围，均无心肺疾病史。本研究经天津医科大学总医院伦理委员会批准，受试者均符合磁共振成像（magnetic resonance imaging, MRI）检查安全标准且对此项研究知情、同意。

### 1.2 图像采集

采用GE 1.5T Twin-speed Infinity with Excite Ⅱ超导型MR扫描仪，8通道心脏相控阵线圈，呼吸和心电门控。采用二维快速稳态进动采集序列（fast imaging employing steady-state acquisition, FIESTA）获得心脏长轴位、短轴位和标准四腔心位图像。短轴位成像参数^[4, 5]^：TR/TE min full/min full，翻转角45°，带宽125 kHz，FOV 35 cm×35 cm，矩阵224×224，扫描层厚/间隔8/0 mm，NEX=1，从心尖到心底共获取9层-13层图像覆盖整个RV，每层扫描时相数为20。采用相位对比（phase-contrast, PC）序列对主肺动脉（main pulmonary artery, MPA）进行扫描并获得幅度图和相位图。成像参数^[5]^：TR/TE自动选择最小重复时间/min full，翻转角20°，带宽31.25 kHz，FOV 40 cm×40 cm，矩阵256×256，NEX=1，扫描时相数为30，流速编码方向为SLICE，速度编码值为150 cm/s。全部检查时间约30 min。

### 1.3 图像分析

将扫描图像传输至GE AW 4.3 MRI工作站，应用Report Card 3.7软件进行数据分析。测量项目如下：

#### 1.3.1 RV形态学和功能指标

在短轴位图像上，连续手动描绘RV收缩末期与舒张末期所有图像的心内膜、心外膜轮廓。软件自动计算出RV舒张末期容积（end-diastolic volume, EDV）、收缩末期容积（end-systolic volume, ESV）、射血分数（ejection fraction, EF）及舒张末期心肌质量（myocardial mass, MM），分别除以体表面积（body surface area, BSA）后，获得RVEDV指数（RVEDV index, RVEDVI）、RVESV指数（RVESV index, RVESVI）、RVMM指数（RVMM index, RVMMI）。在标准四腔心位中间图像上，获得RV舒张末期面积（RV diastolic area, RVDA）、收缩末期面积（RV systolic area, RVSA），并计算RV面积变化分数（RV relative area change, RVFAC），即RVFAC=[(RVDA-RVSA)/RVDA] ×100%^[6]^。在短轴位中间层面图像上，测量并计算获得室间隔曲率（interventricular septal curvature, C_IVS_）、左室游离壁曲率（free wall curvature, C_FW_）及曲率比C_IVS_/C_FW_（图 1）。

**1:**
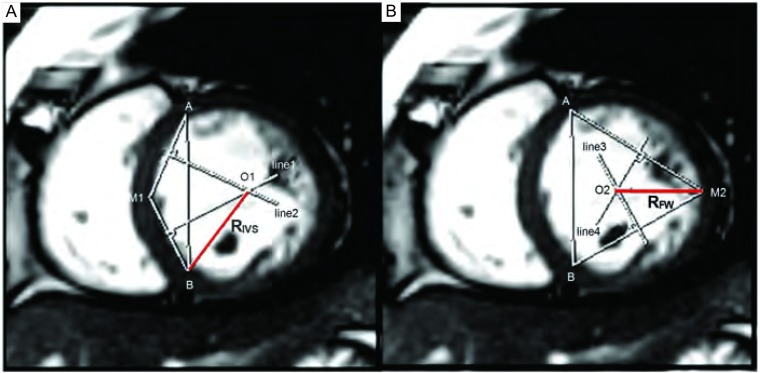
心脏短轴位舒张末期中间层面图像测量曲率。图A、B中A、B两点为插入部，M1、M2两点分别为IVS中点和LV FW中点。A：IVS曲率测量：以A、B、M1三点确定圆O1，其IVS半径记为R_IVS_，IVS曲率记为C_IVS_，C_IVS_=1/R_IVS_。B：FW曲率测量：以A、B、M2三点确定圆O2，其FW半径记为R_FW_，FW曲率记为C_FW_，C_FW_=1/R_FW_ Mid-short axis cine images at the end-diastolic phase for curvature calculation. Two points-junction 1 (A) and junction 2 (B)-are initially positioned at the junctions between the IVS and LV FW. Two additional points are then marked in the middle portion of IVS (M1) and the FW (M2). By considering A, B, and M1, R_IVS_ is derived by applying the three-point circle method, C_IVS_=1/R_IVS_. A, B, and M2 are used similarly to derive the R_FW_, C_FW_=1/R_FW_. IVS: interventricular septal; LV: left ventricle; FW: free wall; R_IVS_: radius of the IVS; R_FW_: radius of the FW. C_IVS_: curvature of IVS; C_FW_: curvature of FW

#### 1.3.2 血液动力学指标

在MPA轴位幅度图中，手动描记MPA血管轮廓内缘，勾画出感兴趣区（region of interest, ROI），追踪一个心动周期各个时相的ROI，并将ROI位置复制到各时相相位图中。由软件计算出MPA在一个心动周期的正向峰值流速、最大截面积和最小截面积，顺应性=[（最大截面积-最小截面积)/最小截面积]×100%^[5, 6]^。

### 1.4 统计学方法

采用SPSS 17.0统计软件对数据进行统计学分析，计量资料符合正态分布以均数±标准差（Mean±SD）表示。PH组与对照组的测量结果采用两独立样本*t*检验进行比较。通过逻辑回归（*Logistic* regression, LR）模型来整合MPA正向峰值流速、顺应性、曲率比、RVFAC在检出PH中的权重并计算出综合评估指标^[7, 8]^，采用受试者工作特征曲线（receiver operating characteristic curve，ROC曲线）计算上述四种指标及综合评估指标曲线下面积（area under the curve, AUC），比较各指标检出PH的灵敏度、特异度和准确度。以*P* < 0.05为差异有统计学意义。

## 2 结果

### 2.1 PH组与对照组RV形态、功能及MPA血液动力学结果比较

与对照组相比，PH组RVEDVI、RVESVI、RVMMI、MPA最大截面积、最小截面积均明显升高（*P* < 0.001）；PH组RVEF、RVFAC、曲率比、MPA正向峰值流速及顺应性均明显低于对照组（*P* < 0.001）（表 1，图 2）。

**1:** PH组与对照组RV功能及MPA血液动力学参数比较 Comparison of RV function and MPA hemodynamic parameters between PH and control group

Parameters	Control group (*n*=22)	PH group (*n*=56)	*P*
RVEDVI (mL/m^2^)	76.44±10.69	132.12±58.75	< 0.001
RVESVI (mL/m^2^)	34.99±6.91	88.33±48.45	< 0.001
RVMMI (g/m^2^)	22.65±4.93	37.97±14.22	< 0.001
RVEF (%)	53.20±6.93	35.94±11.97	< 0.001
RVFAC (%)	42.80±7.89	24.27±12.61	< 0.001
Curvature ratio	0.89±0.10	0.53±0.37	< 0.001
Positive peak velocity (cm/s)	83.32±12.57	60.10±17.87	< 0.001
Maximal area (cm^2^)	6.15±1.27	12.76±4.20	< 0.001
Minimal area (cm^2^)	4.03±0.98	10.73±3.95	< 0.001
Distensibility (%)	54.39±17.54	21.16±12.34	< 0.001
RVEDVI: right ventricle end-diastolic volume index; RVESVI: right ventricle end-systolic volume index; RVMMI: right ventricle myocardial mass index; RVEF: right ventricle ejection fraction; RVFAC: right ventricle fractional area change; PH: pulmonary hypertension.			

**2:**
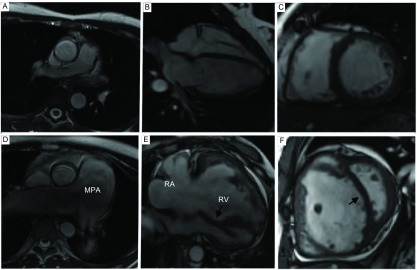
健康志愿者与PH患者CMR图像比较。A-C：女性，27岁，健康志愿者。A-C分别为轴位、四腔心位及短轴位图像。A显示正常MPA。B、C显示正常LV、RV。D-F：女性，28岁，结缔组织病相关性PAH（SLE）。D：轴位图像示MPA明显增粗、扩张。E：四腔心位图像示RV、RA明显扩张，小梁增多，室间隔向左弓（箭头）。F：短轴位图：RV心肌肥厚，心腔扩张，室间隔向左弓（箭头），LV受压变形 PH group and controls comparisons. A-C: Female, 27 yr, Healthy volunteer. FIESTA cine images at axis, four chamber and short axis showed normal MPA, RV and LV. D-F: Female, 28 yr, connective tissue disease associated PAH (SLE). D: Axis image, enlargement and dilatation of the MPA obviously. E: Four chamber cine image, enlargement and hypertrophy of RA and RV, increase in trabecular, 1eftward ventricular septal bowing (arrows). F: Short axis cine image, enlargement and hypertrophy of RV, leftward ventricular septal bowing (arrows), LV deformation. PH: pulmonary hypertension; CMR: cardiac magnetic resonance; MPA: main pulmonary artery; LV: left ventricle; RV: right ventricle; PAH: pulmonary arterial hypertension; SLE: systemic lupus erythematosus; RA: right atrium

### 2.2 CMR指标检出PH的效能比较

经ROC曲线分析，四项指标中，顺应性AUC最大为0.950；综合评估指标（最大正向峰值流速+顺应性+曲率比+RVFAC）对检出PH的效能高于任意单一指标，AUC为0.988（表 2，图 3）。

**2:** CMR指标检出PH的效能比较 Comparison of diagnostic performance in PH between different CMR indices

Parameters (*n*=78)	AUC	Sensitivity	Specificity	Accuracy
Positive peak velocity (cm/s)	0.879	0.732	0.955	0.872
Distensibility (%)	0.950	0.875	0.955	0.897
Curvature ratio (%)	0.847	0.821	0.818	0.820
RVFAC (%)	0.882	0.679	1.000	0.769
Positive peak velocity+Distensibility+Curvature ratio+RVFAC	0.988	0.946	1.000	0.962
AUC: area under the curve; RVFAC: right ventricle fractional area change.				

**3:**
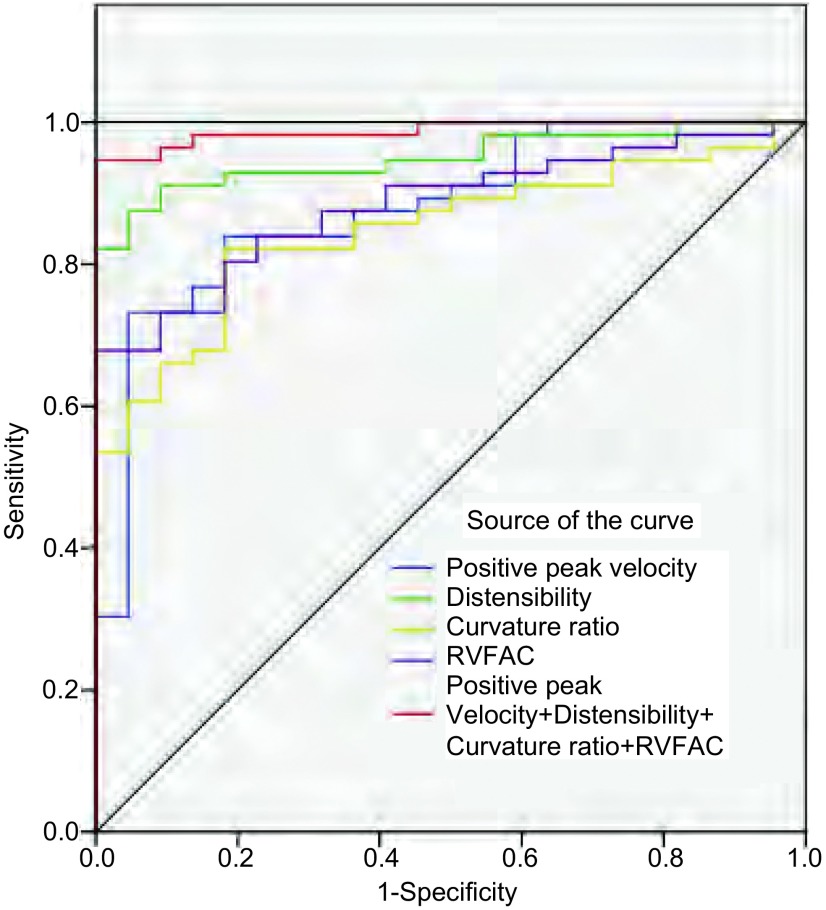
ROC曲线显示CMR指标检测PH异常状态的效能比较 ROC curves showing the diagnostic accuracy of parameters derived-CMR. ROC: receiver operating characteristic curve

## 3 讨论

尽管PH的发病部位在肺血管，但RV功能减退是影响患者预后、活动耐量和生活质量的直接原因^[9]^。因此，以RV-肺循环的整体观念来研究PH尤为重要。CMR作为一种安全、无创的检查，能够准确、可重复地评估RV形态、功能和血液动力学改变，并能直接对伴随的心脏、大血管疾病做出诊断，对PH患者进行“一站式”全面评估。

本研究显示，PH组与对照组相比，RVEDVI、RVESVI、RVMMI均明显高于后者，代表RV扩张、肥厚，而RVEF、RVFAC显著降低，提示RV功能受损，与国内外一些研究^[5, 10]^结果一致。RVFAC为评价RV整体收缩功能的可靠指标，既包含RV纵向功能（如三尖瓣环收缩期位移）又体现横向功能（如室间隔与左室游离壁距离变化），还可间接反映室间隔的运动^[6]^。本研究显示PH组RVFAC明显小于对照组，其原因可能与心肌纤维旋转方向改变导致RV心肌收缩受损有关^[11]^，即横向功能和纵向功能均受到影响，导致RVFAC减小。

曲率亦是评价心脏形态学改变的重要指标，可量化室间隔向左弓形突出的程度。本研究中PH组曲率比明显低于对照组，且预测PH的灵敏度、特异度和准确度分别为82.1%、81.8%、82.0%，AUC为0.874，表明室间隔位置及运动异常，也可用于提示PH。Dellegrottaglie等^[12]^研究显示，曲率比可准确、可重复地预测出右室收缩压>40 mmHg的患者，AUC为0.98，灵敏度、特异度分别为87%、100%，与本研究结果在本质上一致，均证实CMR曲率比可用于评估PH。

PC法可用于评估肺循环血液动力学状态^[13]^。本研究中PH组MPA正向峰值流速、顺应性均明显低于对照组，而MPA最大截面积、最小截面积均明显高于对照组，说明MPA管壁变僵硬，阻力增加，峰值流速减低，均作为评估PH的重要指标^[14]^。顺应性在检出PH中AUC最大为0.950，具有相当高的灵敏度、特异度和准确度，分别为87.5%、95.5%、89.7%。PH患者肺动脉压力升高牵拉血管壁，使可扩张的胶原纤维减少，管壁僵硬，顺应性随之降低^[15]^。

本研究将MPA正向峰值流速、顺应性、曲率比及RVFAC整合计算获得综合评估指标，检出PH效能高于任意单一指标（AUC为0.988），灵敏度、特异度、准确度分别提高至94.6%、100.0%、96.2%。该综合评估指标既包含PH患者RV形态及功能特点，又反映了MPA血液动力学信息，能够更加准确、全面、系统地评价PH患者的病生理状态，具有临床应用价值。

本研究存在的局限性：样本量相对较少，仅包含PAH和CTEPH两个组别的患者。PH组中患者多数为中至重度，且没有进行纵向随访研究。故本研究的结果仍有待进一步充实、验证。

综上所述，CMR能无创、准确地提供PH患者RV形态、功能及MPA血液动力学信息，综合评估MPA正向峰值流速、顺应性、曲率比及RVFAC有利于准确反映PH患者RV-肺循环整体状态，具有临床应用价值。
